# Redox regulation in diabetic kidney disease

**DOI:** 10.1152/ajprenal.00047.2023

**Published:** 2023-06-01

**Authors:** Ilse S. Daehn, Ubong S. Ekperikpe, Krisztian Stadler

**Affiliations:** ^1^Division of Nephrology, Department of Medicine, The Icahn School of Medicine at Mount Sinai, New York, New York, United States; ^2^Department of Pharmacology and Toxicology, University of Mississippi Medical Center, Jackson, Mississippi, United States; ^3^Oxidative Stress and Disease Laboratory, Pennington Biomedical Research Center, Baton Rouge, Louisiana, United States

**Keywords:** diabetic kidney disease, oxidative stress, reactive species, redox

## Abstract

Diabetic kidney disease (DKD) is one of the most devastating complications of diabetes mellitus, where currently there is no cure available. Several important mechanisms contribute to the pathogenesis of this complication, with oxidative stress being one of the key factors. The past decades have seen a large number of publications with various aspects of this topic; however, the specific details of redox regulation in DKD are still unclear. This is partly because redox biology is very complex, coupled with a complex and heterogeneous organ with numerous cell types. Furthermore, often times terms such as “oxidative stress” or reactive oxygen species are used as a general term to cover a wide and rich variety of reactive species and their differing reactions. However, no reactive species are the same, and not all of them are capable of biologically relevant reactions or “redox signaling.” The goal of this review is to provide a biochemical background for an array of specific reactive oxygen species types with varying reactivity and specificity in the kidney as well as highlight some of the advances in redox biology that are paving the way to a better understanding of DKD development and risk.

## Graphical abstract

**Figure:**
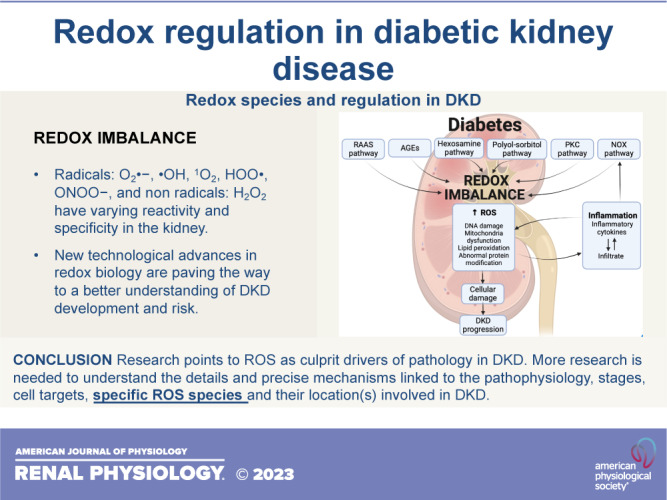

## INTRODUCTION

Diabetes is the leading cause of chronic kidney disease (CKD) and end-stage renal disease worldwide (1–3). About 40% of patients with diabetes have diabetic nephropathy, which is the most common form of CKD (4). Diabetic kidney disease (DKD) is characterized by microalbuminuria, glomerular lesions, and a progressive decline in renal function. To date, there are no drugs that halt or reverse the progression to end-stage renal disease associated with DKD, and typically these patients resort to either dialysis or renal transplantation (5, 6). Understanding the molecular and cellular mechanisms driving disease progression is imperative to the development of more effective therapeutic strategies to address this unmet clinical need.

There is an intricate interplay of pathways that are activated by chronic hyperglycemia during the development of DKD. These include hemodynamic, metabolic, proinflammatory, profibrotic, and oxidative stress pathways (7–10). Over the past decades, growing evidence in the scientific literature has suggested that excessive production of reactive oxygen species (ROS) is a unifying common pathway that links metabolic stress due to hyperglycemia and dyslipidemia with impaired renal hemodynamics (11–17). However, the picture is a lot more complex. First, initially thought to be only deleterious, ROS are also extensively involved in cell signaling and proliferation under physiological conditions (18). Second, ROS represent a diverse set of molecules with different kinetics, reactivity, and compartmentalization. This partially explains the largely unsatisfactory clinical trials with antioxidants in patients with DKD: tackling high levels of free radicals with antioxidants is not necessarily fruitful (19, 20). The approach needs to be fine-tuned, to focus on specific species, their reactions, and potential mechanisms leading to cell death and demise and to distinguish them from those that are, for example, necessary signals to remove injured cells or organelles. In principle, chronic hyperglycemia in diabetes damages renal cells [i.e., mesangial cells, podocytes, glomerular endothelial cells (GECs), and proximal tubular epithelial cells] (12, 21–24) via upregulation of advanced glycation end product-dependent pathways, the hexosamine pathway, the polyol-sorbitol pathway, the protein kinase C (PKC) pathway, the NADPH oxidase (NOX) pathway, and the renin-angiotensin-aldosterone system, all of which are associated with redox signaling in some ways (11, 25–27). Ultimately, these ROS-associated pathways promote glomerular cell dysfunction, renal inflammation, and fibrosis by upregulating DNA damage, mitochondrial dysfunction, lipid peroxidation, and abnormal protein modification (28). These compelling body of work point to ROS as culprit drivers of pathology in DKD, and continuing work is needed to understand the details and precise mechanisms linked to the pathophysiology, stages, cell targets, specific ROS species, and their location(s) involved in renal cell demise.

## REACTIVE SPECIES IN DKD

### Superoxide: a Short-Lived but Important Originator

**Figure 1.**
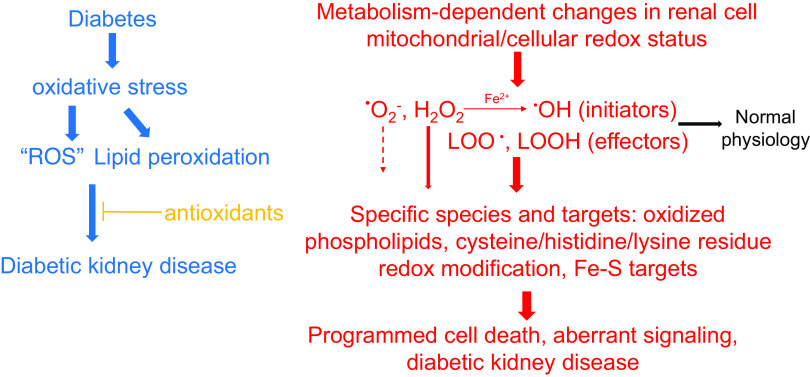
Furthering the concept of oxidative stress and diabetic kidney disease. From a more simplistic view of general “oxidative stress” where many various reactive oxygen species (ROS) molecules were considered similar (*left*, blue), the field is moving to a constantly refined and more specific view of redox molecules and their role in diabetic kidney disease (*right*, red). Although superoxide and the hydroxyl radical are primary species formed in disease pathogenesis, their biochemical reactions may be limited. Differences in the preferred metabolic pathways of various renal cells may also lead to more/less pronounced free radical production. However, the primary radicals are important initiators of downstream reactive species, for example, oxygenated reactive lipids, which are less constrained and yet more specific redox regulators. Novel targets that play a distinct role in programmed cell death (apoptosis and ferroptosis), aberrant signaling due to redox-sensitive residue modification, and renal cell injury can be then identified. H_2_O_2_, hydrogen peroxide; LOO•, lipid peroxide radical; LOOH, (phospho)lipid hydroperoxide; O_2_^•−^, superoxide anion radical; •OH, hydroxyl radical.

Perhaps the most extensive literature in the context of diabetic kidney pathologies and ROS deals with the superoxide anion radical (O_2_^•−^). It is well established and accepted that the superoxide anion radical is an important, primary form of ROS produced by mitochondria and enzymatic extracellular and cytoplasmic sources (29–36). From a biochemical standpoint, as a charged radical (whether of mitochondrial or cytoplasmic origin), superoxide follows strict chemical and kinetic rules that make it rather restricted in its capacity and specificity for a farther-reaching signal or damage. These considerations are often overlooked or simplified. Biochemistry literature from the 1970s onward has described some of these barriers and other considerations. First, the self-dismutation of superoxide is a spontaneous and very fast process (*k* ∼ 10^6^ M^−1^·s^−1^) (37). Second, in the mitochondria, even when produced in large amounts, mitochondrial superoxide dismutase (SOD2 or MnSOD) pulls superoxide away at a nearly diffusion-controlled speed (at a rate constant of *k* = ∼10^9^ M^−1^·s^−1^!) (38). This reaction is three orders of magnitude faster than superoxide production or self-dismutation. This means that superoxide is likely very short lived in most cases and would be converted to H_2_O_2_. H_2_O_2_ is not a radical but is an important secondary cell signaling messenger as it is diffusible. The third barrier is the negative charge of superoxide anion, and therefore superoxide cannot cross membranes due to its charge (with the exception of using voltage-dependent anion channels through the mitochondria outer membrane) (39, 40). In addition, superoxide is actually a reductant and not particularly efficient at oxidizing targets (*E*^0^= −0.33 V) (41). Thus, it might be unreasonable to expect a large, sustained increase in superoxide levels under physiological conditions or even in most pathologies. Although it is very important to recognize that superoxide is a pivotal and a necessary precursor of many further oxidative reactions, we suggest that the general term “ROS” should be revisited from time to time and be separate from superoxide in terms of reactivity and affinity. This will allow the continuous refinement of the role of reactive species in DKD pathogenesis and to define more specifically what ROS is being investigated in a signaling cascade or reaction (Fig. 1).

### Hydroxyl Radical, H_2_O_2_, and the Fenton-Haber-Weiss Reaction

Superoxide can rapidly be dismutated by SOD2 in the mitochondria or cytoplasmic defense mechanism as well (SOD1). The product from this reaction is H_2_O_2_, which is able to diffuse through membranes and is a more efficient and stronger oxidant than superoxide (*E*^0^ = 1.78 V) (42). It is known to exert biological effects as a secondary messenger (43, 44), with relevance to diabetes and insulin resistance (43). H_2_O_2_ in the cytosol is removed by catalase, although there are other enzymatic defense systems against H_2_O_2_ such as peroxiredoxins, thioredoxin, and glutathione peroxidase 1 (45, 46). When the metabolism of a cell changes (by metabolic disease or DKD), the result is often insufficient removal of H_2_O_2_. Excess H_2_O_2_ can react with transition metals like iron (Fe^2+^) if it is available in the ferrous state (47, 48). This reaction, known as the Fenton reaction (which has a rather slow rate constant, *k* ∼ 40–80 M^−1^·s^−1^), produces the hydroxyl radical (•OH), which is extremely short lived, but compared with superoxide, hydroxyl radicals are a much more potent initiator of free radical reactions (for instance, lipid peroxidation) (49). The Haber-Weiss cycle is a two-step reaction where the ferric ion (Fe^3+^) can be reduced back to the ferrous state (Fe^2+^) by reacting with superoxide. Superoxide, in turn, reacts with H_2_O_2_ to form OH^–^ and •OH, converting ferrous iron back to ferric ion. To appreciate this reaction from a biological (and kidney disease) standpoint, one should always consider the presence of Fe^2+^ and Fe^3+^ as well as pH in a given system/cell/experimental setup.

### Singlet Oxygen

Singlet oxygen (^1^O_2_), the lowest excited electronic state of molecular oxygen, is highly reactive. Singlet oxygen reacts with organic molecules, producing peroxide as an initial product and precursor to other products in cell systems, and readily cleaves to yield oxygen-based radicals (e.g., •OH from hydroperoxide) (50). It is most commonly recognized as a product of photochemical reaction in plants and thus perhaps is less relevant in mammalian models and disease compared with other ROS. It is, however, capable of eliciting cellular stress responses in mammalian cells through the activation of mitogen-activated protein kinases (51) or inducing mutations that are not random but mostly targeted at guanine residues (52). Some reports have shown that singlet oxygen is produced during inflammatory and biochemical reactions (53), and levels of the ^1^O_2_-specific oxidation products 10- and 12-(*Z*,*E*)-hydroxyoctadecadienoic acids have been shown to be elevated in a mouse model of type 2 diabetes mellitus (54) as well as in the plasma of patients with early diabetes (55). These findings suggest that ^1^O_2_ may contribute to the regulation of physiological and pathological conditions in mammals; however, more work in this space will provide important insights on this emerging filed.

### Lipid Peroxides and Specific Oxygenated Phospholipids as Messengers

Oxidation/peroxidation of a large group of diverse molecules, phospholipids, leads to the formation of less constrained and more diffusible lipid/phospholipid peroxides, which are reactive molecules with a much longer half-life. As discussed earlier, the hydroxyl radical produced in the Fenton reaction is a potent initiator of nonenzymatic lipid peroxidation. In contrast, enzymatic lipid peroxidation is driven largely by the lipoxygenase enzyme family (as well as cyclooxygenase and some cytochrome *P*-450 enzymes), which use polyunsaturated fatty acids and their esterified derivatives and generate peroxidized products (56). Due to the catalytic site and other factors, this process is a lot more selective than nonenzymatic peroxidation and specific in the products it generates. Which of these two mechanisms is most prominent in CKD/DKD as well as what products they produce that are then linked to cell death are both significant questions. Lipid peroxidation and its basic mechanisms in biology are well established (57), as well as the associated increases in lipid peroxidation, and uncontrolled lipid peroxidation leads to pathology in diabetes and kidney disease (41, 57). However, these redox-active molecules, under the umbrella of lipid peroxides, are very diverse. As phospholipids are the core part of all biomembranes, oxidation of these molecules opens the possibility to a diverse, distinct, and rich variety of lipid-lipid or lipid-protein interactions. Questions arise as to what would be the advantages of a lipid peroxide redox signal over H_2_O_2_ or superoxide? First, not every (phospho)lipid hydroperoxide (LOOH) is capable of redox signaling or becoming a “cell death” signal. This specificity of LOOH can be harnessed to focus on redox regulatory mechanisms controlling cell function and fate in contrast to a random and poorly controlled “ROS” free radical reaction. Second, in contrast to superoxide or H_2_O_2_, many oxidized phospholipids are electrophilic in nature, which gives them a unique biochemical attribute: to have preferential reaction affinity to target protein residues, in the cysteine ≫ histidine ≫ lysine order (58, 59). Due to this distinct reactivity (electrophilic-nucleophilic), the pool of plausible targets could be better defined and a whole new set of molecular targets can be identified. Finally, some oxidized phospholipids, such as oxidized phosphatidylserine, for example, can be externalized by the cell (60, 61), Thus, they are ideal candidates for “marking” damaged cell membranes as autophagy/mitophagy targets or for initiating programmed cell death.

There is an emerging new concept in redox biology about the role and mechanisms of action of these very specific molecules. Efforts to introduce these selective lipid peroxidation mechanisms in the context of kidney diseases would be important to interrogate whether some of these specific oxidized molecules could be predictive biomarkers of cell death programs in CKD or DKD and in which part of the nephron/what renal cell are they prominent. Indeed, many of our own results and the results from others have suggested increased lipid peroxide levels in DKD models (streptozotocin-induced rats, *db*/*db* mice, and the high-fat diet-induced obesity model) (62–64). Our recent discoveries showed that there are specific oxidized phospholipid species (oxidized phosphatidylethanolamine and lysophosphatidylenthanolamine) that are increased in the kidneys of diabetic mice (63). Oxidized phosphatidylethanolamines have recently been proposed as specific cell death signals in the literature (61, 65, 66) and thus plausible candidates to be causative to renal injury. One group has recently linked ferroptosis, a type of programmed cell death dependent on iron and characterized by the accumulation of LOOH, to acute kidney failure (67) using the ablation of glutathione peroxidase 4, which is a unique enzyme capable of removing oxidized membrane phospholipids, indicating that ferroptosis-driven machinery likely exists in injured renal cells. Lipid peroxidation was not confined to the mitochondria but spread through cell membranes, and mice developed renal failure (68). The ferroptosis phenomenon that triggers a “wave of death” in kidney tubular injury (69) can be inhibited by ferrostatin-1, which was shown to preserve renal function and decreased histological injury, oxidative stress, and tubular cell death in a folic acid-induced acute kidney injury (AKI) model (70) and was shown to inhibit lipid peroxidation but not mitochondrial ROS formation or lysosomal membrane permeability in AKI (71). However, the contribution of specific oxidized phospholipids as redox signals to DKD/CKD pathogenesis has not been established and remains an open and intriguing question.

### Nitric Oxide and Peroxynitrite

Nitric oxide (NO) is probably the most well-known gasotransmitter molecule that is also a free radical. Originally discovered as the endothelium-derived relaxing factor, NO is an essential signaling molecule, a vasodilator, and a modulator of redox cascades, and, importantly, it has been shown to regulate renal cell function. NO is localized in the cytoplasm and mitochondria. Produced from l-arginine, where the enzyme NO synthase (NOS) catalyzes the reaction with oxygen, NO is a pivotal messenger molecule that can freely diffuse across biological membranes. It has been extensively studied as it is a classical “double-edged sword” molecule in physiology and pathology, as lack of NO is detrimental to the vasodilation process and blood pressure control (72); if overproduced by the inducible form of NOS, NO can trigger proinflammatory reactions (73, 74). Simultaneous excessive production of superoxide and NO can lead to the formation of peroxynitrite (ONOO^−^), an extremely potent reactive species, in a rapid, nearly diffusion-controlled reaction, although peroxynitrite is not able to travel long distances as it is rapidly protonated. At the production site and at a few molecules distance, however, ONOO^−^ can do extensive damage, for example, by nitrating protein residues on tyrosine. ONOO^−^ has been linked to several complications where it can trigger cell death and downstream mechanisms through poly(ADP-ribose) polymerase activation, such as diabetic neuropathy and DKD (75–77). Thus, NO has a complex role in complications of diabetes, including in the kidney.

NO in the kidney can be produced by various renal cells (e.g., endothelial cells, the macula densa, or podocytes of the glomerulus) upon a number of stimuli such as angiotensin II, increased tubular perfusion, or insulin (78–80). The role of NO in the glomerulus is still an understudied area in DKD. Studies have suggested that NO bioavailability is reduced in the kidney, including in glomerular cells during renal pathogenesis and that restoring levels of NO is beneficial for normal function (79). For example, when endothelial (eNOS or NOS3) is deleted in the *db*/*db* mouse model, renal pathology is greatly exacerbated, and diabetic nephropathy is accelerated, with the model presenting endothelial dysfunction, glomerular damage, and albuminuria (81). In the context of diabetes, high glucose in podocyte culture combined with low NO levels alters podocyte actin cytoskeleton behavior and dynamics (82). Much less is known about the details of NO signaling in the glomerulus and podocytes (80), for example, how increased Ca^2+^ signaling is connected to NO regulation in a disease setting.

## SOURCES OF REACTIVE SPECIES: CELLULAR, MITOCHONDRIAL, AND ENZYMATIC

### NADPH Oxidases

One of the most well recognized and important cytoplasmic sources of ROS, namely, superoxide, is the enzyme NOX. NOXs catalyze the electron transfer reaction of NADPH with oxygen and the production of superoxide (83). The family has several isoforms, including NOX1–NOX5. They are extensively studied because of their unique function of producing superoxide under physiological conditions and thus having a “dual role.” Precise regulation of NOX, therefore, is important to maintain redox balance as in the kidney, and the NOX4 isoform seems to be especially important in the context of DKD and other renal diseases. Although it is important to recognize that NOX4-derived oxidants (e.g., H_2_O_2_) modulate important cell functions, excessive expression and ROS production can induce cell death pathways and contribute to cellular injury (84). NOX4 upregulation has been linked to DKD as well as to polycystic kidney disease and hypertensive CKD (85–87). It has also been proposed that oxidant molecules produced by NOX4 can lead to depolarization of the mitochondrial membrane, affecting mitochondrial potential and thus exacerbating mitochondrial damage (88). The induction of diabetes in a podocyte-specific NOX4 ablated model led to less severe DKD as demonstrated by attenuation of proteinuria and glomerular basement membrane thickening (89). In contrast, NOX4 ablation in tubular cells was dispensable in DKD, highlighting the importance of cell type and compartment specificity when studying redox regulation (90). However, as NOX4 is constitutively active, a complete absence of NOX4 is also undesired as it leads to kidney fibrosis (91). NOX2 has also been shown, albeit to a somewhat lesser degree, to contribute to redox-mediated renal injury (92). With regard to other isoforms of NOX, initially, there was a paucity in research in the kidney in DKD. More recently, however, it has been shown that NOX5 accelerates renal injury in diabetic models (93, 94). NOX5 is also expressed in human, but not in mouse, glomeruli and seems to be increased in diabetics (95). NOX1, on the other hand, does not seem to play a role in DKD pathogenesis (96).

### Nitric Oxide Synthases

NOSs are the main enzymatic source of NO. They catalyze the reaction of NO formation using the substrate l-arginine (NO can also be formed from nitrate without the enzymes, e.g., during hypoxia). The three isoforms, originally named by their place of discovery, are neuronal NOS (nNOS or NOS1), endothelial NOS (eNOS or NOS3), and inducible NOS (iNOS or NOS2). Although the first two isoforms are constitutionally expressed and regulated by Ca^2+^, iNOS is typically upregulated severalfold in inflammatory processes and can produce large amounts of NO (97–99). All three isoforms are present in the kidney, where they play a role not only in blood pressure control but also in several other processes including, but not limited to, renal Na^+^ handling or redox balance, as we described in *Nitric Oxide and Peroxynitrite*. Limitation of the substrate (l-arginine) availability in DKD can also be a further factor in disease progression and kidney damage (100). NOS can also produce superoxide radical. An altered superoxide/NO balance then can alter NO bioavailability, contributing to or further exaggerating pathological conditions in the kidney, including hypertension and the progression of DKD. Deficiency of the cofactor tetrahydrobiopterin (BH_4_) can also result in NOS uncoupling, undesired free radical production, and the aforementioned reactions of peroxynitrite.

### Xanthine Oxidoreductase

Excess ROS in diabetes have been also attributed to increased xanthine oxidoreductase (XOR) activity (101). XOR refers to both xanthine dehydrogenase (XDH) and xanthine oxidase (XO), interchangeable forms of the same enzyme encoded by the *XDH* gene. XDH is reversibly converted to XO by an oxidation-sensitive modification of cysteine residues (102). Another considerable contributor of XDH to XO conversion is limited proteolysis, which is not reversible. As an example, XDH released in circulation is rapidly converted to XO through plasma proteases; thus, almost all XOR in the blood is XO. XDH catalyzes the oxidation of purine substrates (xanthine and hypoxanthine) and use NAD^+^ as an electron acceptor, whereas XO uses O_2_ as an electron acceptor and generates H_2_O_2_ and $O_{2}^{-}$; the reaction is a major enzymatic source of cellular and extracellular ROS (101, 103–105). Uric acid is the end point of purine metabolism in humans, whereas allantoin is the end point in most mammals. In humans, high XOR levels have been demonstrated to be a risk factor for cardiovascular and kidney diseases, including DKD (106–108), and both hyperuricemia and hypouricemia can have negative consequences for renal health (103, 109).

We have recently demonstrated that DKD predisposition in mice was attributed to a variant regulating XOR activity and a higher redox state in diabetes (110). We also uncovered promoter *XOR* ortholog variants in humans associated with a high risk for DKD and other diabetic complications. Another study showed that XOR inhibition could attenuate oxidative stress and protect against DKD by inhibiting the VEGF-NOX signaling pathway in an animal model of DKD and human GECs (111). Increased XOR in streptozotocin-induced DKD was associated with increased VEGF/VEGF receptor 1 and VEGF receptor 3 levels in the kidneys, followed by the activation of NOX1, NOX2, and NOX4 expression and by FoxO3a phosphorylation and activation of eNOS. In our study, we demonstrated that XOR inhibition prevented mitochondrial oxidative DNA damage (110); hence, a potential feedforward interplay between XOR-derived ROS enhancing mitochondria-derived ROS release could provide a mechanism that promotes a vicious cycle of sustained stress and accumulation of oxidized products. Understanding these interactions is important, as they are derived through various sources of ROS in DKD; hence, studies assessing the contribution of redox processes in DKD are expected to provide valuable tools in DKD therapy in the upcoming years.

### Mitochondria as a Source of ROS

As we have previously mentioned, there is ample evidence that superoxide is a major primary free radical produced in the mitochondria besides other cellular sources, whether it be the kidney or other organs (29, 36, 112). The mitochondrial electron transport chain (ETC) always leaks some superoxide, especially at the Q level, which can be potentiated under certain conditions or largely dissipated by collapsing the membrane potential. Overproduction of mitochondrial superoxide as a main form of oxidant stress and a primary event in the pathogenesis of DKD has been proposed in several studies (26, 113, 114). The consensus, however, is not clear on whether increased mitochondrial superoxide is a prominent driver in DKD and whether it has far-reaching effects or more of a role in local mitochondrial damage leading to a cascade of events. There is no doubt that, indeed, diabetes causes mitochondrial impairments where increased superoxide can damage ETC protein complexes or lead to alterations in electron transport. Accumulation of misfolded molecules due to oxidative damage and cytochrome *c* release can trigger cell death cascades (115, 116). Mitochondrial superoxide, however, is unlikely to impact mitochondrial DNA. This is because superoxide with a negative charge is unlikely to be the seminal biochemical species to interact with mitochondrial DNA with a similarly negative charge, although this concept somehow seems to be popular. It is more likely that the resulting H_2_O_2_ as a downstream product is the contributor in deleterious mitochondrial reactions. More recent studies using transgenic approaches with application of a redox-sensitive GFP biosensor in *db*/*db* mice have suggested that mitochondrial superoxide in DKD is produced at the complex I level (117). There is a link between mitochondrial dynamics such as mitochondrial fission, mitochondrial biogenesis (118, 119), and mitochondrial superoxide as well. Interestingly, there are also contrasting reports on the levels of mitochondrial superoxide in DKD models from other prominent groups (120, 121), proposing decreased levels and a role for AMP-activated protein kinase (AMPK) in the process. Certainly, differences in the models used, timing, compartmentalization in cells, methodological approaches using fluorescent dyes versus other fluorescent reporter-based methods, or electron paramagnetic resonance (EPR) spectroscopy could all contribute to the contrasting results.

We propose, however, that, based on the kinetic considerations and biochemical restrictions discussed earlier, perhaps mitochondrial superoxide, even if increased in pathological models, can neither have more than local effects nor can it escape mitochondria much. Its role as a “starter” to further reactions, including the Fenton reaction and subsequent lipid peroxidation initiation by the resultant hydroxyl radical, however, could be key to initiate a cascade, including reactive lipids and even H_2_O_2_ formation that are longer-lived and farther-reaching species. Mitochondria can be ideal places for such reactions because of the proximity of the lipid bilayers and phospholipids in membranes to the ETC where ROS are formed. The ferrous state iron necessary for the Fenton reaction could come from damaged Fe-S complexes of the ETC; however, it has yet to be established whether the reduced/oxidized state of each complex changes significantly during the pathogenesis of DKD. Some of our unpublished observations using low-temperature EPR indicate that there are damaged ETC complexes/proteins in the kidney of diabetic models (complex I and aconitase in *db*/*db* mice) that could provide Fe^2+^ to drive the Fenton reaction. Regardless, further studies are needed to clarify the role of mitochondrial oxidative stress and the exact molecular identity of species participating in the development and contributing to the progression of DKD.

## GLOMERULAR OXIDATIVE STRESS

In DKD, hyperglycemia and dyslipidemia can disturb the cellular redox balance, especially in glomerular cells. The glomerulus consists of four different cell types: parietal epithelial cells, mesangial cells, podocytes (visceral epithelial cells), and GECs, which cover the luminal surface of glomerular capillaries and are the cells of the glomerulus in direct contact with the blood. Podocytes wrap around the glomerular capillary vessels by an elaborate net of interdigitating foot processes, which bear slit diaphragm proteins (nephrin and podocin) between the foot processes to form a size-selective barrier (122). GECs and podocytes share a common extracellular matrix, the glomerular basement membrane, and together they form an interconnected glomerular filtration barrier. The diabetic milieu can trigger oxidative stress responses in these cells that have been suggested to be one of the key pathways in the initiation and progression of DKD.

Podocytes have been studied extensively as primary targets in DKD, as their depletion from glomeruli is strongly associated with the progression of DKD and the breakdown of the glomerular filtration barrier (123). For instance, the addition of high extracellular glucose (30 mmol/L) was shown to rapidly stimulate intracellular ROS generation of conditionally immortalized podocytes via NOX (124). Inhibition of NOX4 reduced high glucose-elicited ROS generation and upregulation of profibrotic markers of the podocyte (89). Overexpression of NOX5 in podocytes was shown to promote albuminuria, podocyte foot process effacement, and an elevation of systolic blood pressure through the generation of ROS, which was further aggravated with diabetes (93).

Studies have shown the critical role of mitochondria in the development and progression of glomerular injury as well (43, 125), and the role of mitochondria and mitochondria superoxide in podocytes has been recently reviewed (126). It has been demonstrated in podocytes that there is an excess of mitochondrial fission in podocytes via increased Rho-associated coiled-coil-containing protein kinase 1 activity (127, 128), and there is altered mitochondrial lipid composition in podocytes (125) as well as increased mitochondrial superoxide through a reduction of complex I and III activity (117) or through enhanced expression of p66Shc in podocytes in a diabetic setting (127, 129). The role of XORs in podocytes has also been explored, where topiroxostat, a nonpurine, selective XO inhibitor, was shown to ameliorate proteinuria by preserving podocyte functional molecules such as nephrin, podocin, and podoplanin (130).

Mesangial cell proliferation and matrix deposition are key features of DKD that contribute to glomerulosclerosis. Research over the years has demonstrated that high glucose induces mesanglial proliferation, and this could be mediated through NOX activity-derived ROS (131). Also, mesangial cells treated with high glucose increased ROS production, decreased SOD activity and glutathione levels, upregulated p53 expression, and initiated programmed cell death (132). Altered extracellular matrix proteins in a diabetic setting was also shown to increase intracellular oxidative stress of mesangial cells, leading to progressive glomerular damage (133). Although mesangials cells are key players in the onset and progression of DKD, the ROS-specific roles are less understood in pathogenesis.

Another area where there are relatively few studies exploring ROS production in DKD is in GECs (134); however, research from our laboratory demonstrated that diabetic milieu (high glucose or serum from diabetic mice)-mediated GEC mitochondrial oxidative stress and impaired autophagy resulted in oxidatively damaged DNA accumulation in vitro, whereas exposure of podocytes to the same diabetic milieu resulted in minimal oxidative stress (115). This area of investigation highlights the unique cell-specific responses and activation of coping mechanisms triggered by oxidative stress. In vivo, we have also shown that genes involved in oxidative phosphorylation and mitochondrial dysfunction were the most enriched in a transcriptomic comparison of mice susceptible and resistant to DKD (135). Importantly, mitochondrial oxidative stress and DNA damage in DKD-susceptible mice was specific to endothelial cells, resulting in a loss of endothelial fenestrae and subsequent podocyte depletion, which was prevented with treatment with the mitochondrial scavenger mitoTEMPO, which also has alkyl radical scavenging properties (135, 136). It is again worth noting that while mitoTEMPO blocks the initiating species (superoxide), it is likely that H_2_O_2_ or other more mobile and longer-lasting reactive species are causal. Other studies have confirmed that the mRNA profiles of isolated GECs from diabetic mouse kidneys demonstrated distinct upregulated pathways involving mitochondrial function and oxidative stress. Meanwhile, changes in the regulation of actin cytoskeleton-related genes were the major pathways affected in podocytes isolated from the same diabetic mice (137, 138). In our study, we showed that factors secreted by stressed GECs could mediate podocyte apoptosis, whereas the effect was blocked by treating GECs with mitoTEMPO (115). These findings suggest that increased endothelial cell mitochondrial ROS production by exposure to diabetic milieu could mediate podocyte injury and therefore breakdown of the glomerular filtration barrier through intercellular cross talk.

## PROXIMAL TUBULES

The proximal tubules comprise the vast majority of the kidney cortex. They are metabolically very active and thus very rich in mitochondria to provide ATP for the large energy need of Na^+^ pumps. Their high energy need is primarily covered from mitochondrial fatty acid oxidation (139–141). Their glycolytic ability is not significant, with the exception of the S3 segment. Although excess fatty acid metabolites can be shuttled to cytoplasmic lipid droplets to shield from toxicity, this buffer capacity is limited in proximal tubular cells (PTCs) (142, 143). Therefore, any disturbance in fatty acid oxidation can cause PTC atrophy through apoptosis (15, 144–149). Ultimately, the cells are replaced with scar and fibrous matrix proteins (150). This leads to tubular cell/interstitial fibrosis, which is characteristic of almost all end-stage renal diseases (128, 151, 152), including those stemming from diabetes. Because PTCs are so heavily reliant on mitochondria, it is also then logical to propose that alterations in mitochondrial metabolism and bioenergetics during DKD will cause a significant change in redox balance and oxidative stress in PTCs. Discoveries from a multiconsortium team showed that metabolic alterations in tissues affected by diabetic complications are tissue specific, with increased fatty acid metabolism in the kidneys of type 2 diabetic mice and humans (153). Increased β-oxidation, however, is not matched with ATP production. The kidney cortex (∼90% tubular cells) shows accumulation of intermediate metabolic products from mitochondrial β-oxidation. After an initial increase, tricarboxylic acid cycle activity slows down in advanced kidney disease, and organic acid metabolites from the tricarboxylic acid cycle are lost into the urine. These would suggest a lipid/substrate overload scenario in the PTC. Consistent with these studies, our own work showed incomplete mitochondrial fatty acid oxidation causing PTC demise (154, 155) in our mouse models of CKD. Incomplete fatty acid oxidation then fosters a microenvironment conducive to oxidant stress (156). This is because a chronic increase in nutrient stress and substrate entry to the mitochondria produces reducing equivalents in excess of that which can be handled by the ETC. This leads to increased NADH and FADH_2_ levels and tips the NAD^+^/NADH balance (157). Such an increase was proposed to induce electron “backpressure” and ROS production. Indeed, we found increased lipid peroxide radical formation detected by gold standard EPR spectroscopy in our models (154), and, in agreement, others observed either increased lipid peroxidation or other forms of ROS species in various mouse models of DKD, high-fat diet-induced CKD, or STZ-induced diabetes (158–162). There is also evidence showing that mitochondria targeted small-molecule compounds with redox/antioxidant properties largely restore PTC morphology and function (163, 164), suggesting that protecting mitochondria-rich PTCs through a redox-based approach can be beneficial in DKD.

Although PTCs have very little glycolytic activity in a normal kidney, the majority of glucose is reabsorbed in proximal tubules. This occurs through the Na^+^-glucose cotransporter (type 2; SGLT2) located at the PTC brush border. SGLT2 thus has a pivotal role in both osmolarity maintenance and glucose transport. Dysfunction of SGLT2 in diabetes can impact not only glucose metabolism but also might be relevant for redox balance as well. How could this occur? In DKD, SGLT2 is overactive, causing excessive glucose and Na^+^ reabsorption. The energy (ATP) need of the transporter is substantial, and thus its impact on mitochondrial function and potentially increased ROS generation is not negligible. Both high glucose and higher ROS then can further exacerbate SGLT2 overactivation. It is then possible that SGLT2 inhibitors (which have recently been the focus of numerous animal and human studies in DKD) have additional beneficial effects on the redox environment as well. SGLT2 inhibitors, such as empagliflozin, canagliflozin, and similar, are drugs targeting SGLT2, leading to glucosuria. Besides affecting blood glucose levels and improving insulin resistance, the beneficial effects in diabetic neuropathy and delaying progression of DKD pathogenesis are now obvious in light of the current literature. Indeed, some of the studies already have demonstrated improvements in redox parameters as well. SGLT2 inhibitors have been shown to improve SOD and MnSOD activity in patients (165). Dapagliflozin reduced urinary excretion of the DNA oxidation marker 8-oxo-dG (166). Empagliflozin has been shown to reduce superoxide production and to increase glutathione content (which is a major cytoplasmic redox defense molecule besides many other functions) and catalase expression in leukocytes from diabetics (167). Others have shown beneficial effects with regards to the earlier discussed NOX enzymes in early DKD in the *db*/*db* model (168). Interestingly, a study also found increased lipid peroxidation markers after SGLT2 inhibitor treatment in patients with diabetes, suggesting that ketogenesis induced by the inhibitor may also increase lipid peroxidation (169). Blockade of SGLT2 using molecular biology tools (e.g., siRNAs) relieves oxidative stress in various kidney cell types, including tubular and mesangial cells (170, 171). Thus, it seems that SGLT2 inhibition in general also leads to improvements in redox balance in DKD and may further benefit patients beyond direct effects of transport inhibition. The open question is thus: is this simply because of a secondary effect of reducing hyperglycemia or rather there is activation of pathways and mechanisms directly impacting redox signaling mechanisms even in cells that do not express the transporters? Some beneficial effects of SGLT2 inhibitors have been demonstrated in nondiabetic scenarios as well (e.g., cancer cell proliferation) (172), so it is possible that normalizing cellular energy homeostasis and affecting redox balance adds to the already known effects of these drugs.

Importantly, some of the current approaches (such as fibrates) are not ideal for patients with DKD from a redox perspective. Not only do they elevate serum creatinine levels by ∼15–20%, which is not ideal for diabetics, but accelerating fatty acid metabolism in the mitochondria may also lead to undesired effects such as further ROS production (157). This can be counterintuitive in an already obese patient with diabetes due to the formation of enhanced advanced glycation end products from ROS. SGLT2 inhibitors thus may bring multiple benefits at once.

## REDOX REGULATORY MECHANISMS IN DKD

**Figure 2.**
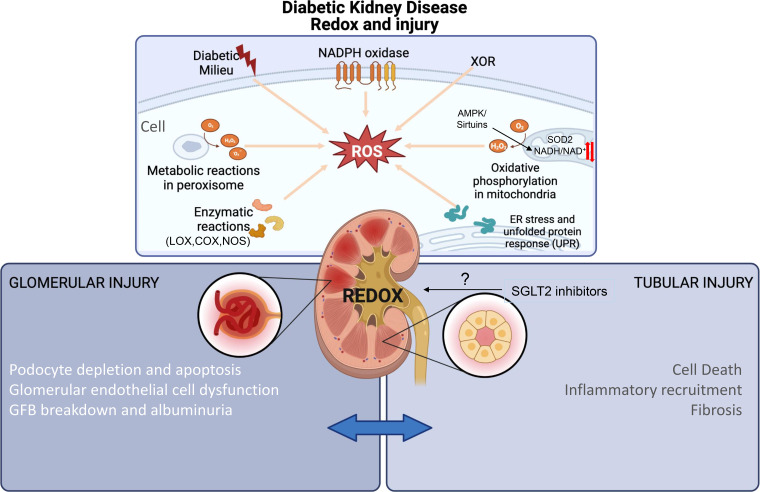
The complexity of redox regulatory pathways in diabetic kidney disease. Some of the many specific redox regulatory players in diabetic kidney disease are highlighted herein for a summary. “Reactive oxygen species” (“ROS”) is used here to cover the many different species that may be formed from the various pathways, but it is always recommended to define what exact species is measured or proposed in a given pathway. AMPK, AMP-activated protein kinase; COX, cyclooxygenase; LOX, lipoxigenases; NAD(H), nicotinamide adenine dinucleotide; NOS, nitric oxide synthases; NOX, NADPH oxidases; SGLT2, Na^+^-glucose transporter 2; Sod2, superoxide dismutase 2; XOR, xanthine oxidoreductases.

Importantly, although it has been well established that redox dyshomeostasis and DKD are connected, several concepts still somewhat lack detail and specificity. This is partially due to the initially oversimplified concept of free radicals versus antioxidant balance. In reality, such a complex and rich variety of redox molecules and their reactions cannot be simplified into a singular concept. This may be also partially the reason why most of the previous clinical trials with “antioxidants” led to disappointing results, not just in DKD but also in diabetes and for other diabetic complications in general. More understanding on the specific regulatory function of specific redox signaling molecules is needed. The molecular entity, production site, biochemical properties, and possible redox reactions in vivo are still unclear and need to be better defined and understood. Below, we wish to highlight and examine a few of many of the proposed redox regulatory mechanisms in DKD and provide some new insights as well for future approaches (Fig. 2).

An intriguing possibility is to consider reactive electrophile lipids and oxygenated (phospho)lipids as unique candidates for redox messenger molecules in DKD. First, their biochemical property, namely, the electrophile-nucleophile reaction, enables them to be a more selective and targeted redox signal, even if they are present at low concentrations. Unlike uncontrolled/poorly controlled or confined reactions of other ROS, electrophile lipid peroxidation end products react with nucleophile protein residues such as the amino acid residues cysteine, histidine, or lysine (58). Second, many of these oxidized phospholipid molecules are externalized by the mitochondria or cell and thus can “mark” and flag cells and membranes as autophagy/mitophagy targets for the elimination process. There is a large body of literature available now that discusses specific roles for electrophilic reactive lipids in various diseases (for a review, see Ref. 58). In a chronic disease like DKD, which develops slowly, it is logical to propose a redox process that is able to produce and maintain distant effects over space and time. Our previous work in DKD demonstrated that when these electrophilic lipid peroxidation products are generated in podocytes, they react with redox-sensitive cysteine residues of RhoA, an important small G protein controlling podocyte cytoskeleton dynamics, motility, and foot processes. When the cysteine residues were mutated to alanine, RhoA was functional but lost its redox activity, and podocyte motility was normal despite the presence of lipid peroxides (173). These observations again suggest a specific redox signaling role for electrophilic lipids instead of just general toxicity or “oxidative stress.” Given the diverse cellular landscape of the kidney, it would be interesting to see in the future which renal cells metabolically favor reactive lipid peroxide production and what are the molecular targets of those ones that are increased during the progression of DKD.

Redox regulation and maintaining redox balance in the kidney also appears to be connected to a master regulator of energy sensing, AMPK. This is an intriguing area of research because of the obvious energy and nutritional imbalance in diabetes and its connection to redox processes. In principle, AMPK activity has been shown to be reduced in DKD in animal models (120). Why it is exactly reduced is still somewhat unclear but perhaps relates to the fact that AMPK is very sensitive to small changes in nutrient status, nutrient stress, and also a chronically overfed state. AMPK activation is known to be beneficial in obesity- and diabetes-related nephropathies (120, 158, 174). With regard to modulating redox homeostasis, AMPK seems to reduce NOX4 activation and consequent superoxide and H_2_O_2_ emission. In conjunction with AMPK, there are downstream regulators of mitochondrial function and biogenesis that are also relevant in redox biology, such as sirtuins or mammalian target of rapamycin (mTOR). Sirtuins are NAD^+^-dependent deacetylases; thus, they are coupled with the maintenance of NAD^+^ levels and NADH-to-NAD ratios. In diabetic glomeruli, mTOR has been shown to be activated, and, importantly, NOX4 has also been shown to stimulate mTOR (175). Thus, NOX4 and mTOR stimulation and redox regulation are intertwined. Stimulation of AMPK and sirtuin has been shown to be beneficial in the diabetic *db*/*db* mouse kidney disease model (176). In conjunction with AMPK and mitochondrial oxidative stress, we also have to mention the mitohormesis theory. Briefly, the hormesis view proposes that not only overproduction of mitochondrial superoxide is harmful but also that too little superoxide can also be damaging as cells require a basal level of ROS for proper cellular function and survival (121). In the kidney, mitochondrial superoxide likely regulates many important processes and signaling pathways that are essential for the normal function of redox-sensitive moieties and cell activity. The seemingly contrasting studies in the literature can be then brought to an agreement: a departure from a nearly five-decade-old, more simplistic view toward a more context-dependent and specific approach will likely bring us closer to understand redox regulatory pathways in the diabetic kidney, whether it is early stage of the disease, progressing DKD, or whether we are focusing on glomeruli, podocytes, tubular cells, or other nephron segments and cell types.

NADH/NAD^+^ balance is another central tenet in redox homeostasis in cells in general. Although these molecules were initially thought to be simply cofactors and electron carriers, literature data are mounting now demonstrating how important the NADH-to-NAD^+^ ratio is in several chronic and acute diseases, not limited to the kidney. A change in cell metabolism, oversupply of substrates leading to NADH production, or, conversely, pathways consuming NAD^+^ can contribute to dysfunction in the ETC and can tip the NADH-to-NAD^+^ ratio. From a redox view point, this can lead to electron “leak” (as not all excess NADH will be used to make ATP) and backpressure leading to more ROS production. With regard to type 2 diabetes and related DKD, an oversupply of nutrients without ATP need likely alters the ratio in favor of too much NADH, which can lead to electron backpressure and excessive formation of mitochondrial superoxide. Besides diabetes, a similar concept has been shown to be essential in AKI as well, where the acute injury triggers excess NAD^+^ consumption and alters the NADH-to-NAD^+^ ratio (177).

Finally, mitochondrial dynamics, such as mitochondrial fission and fusion and redox homeostasis in the kidney, have also been demonstrated to be interconnected. Disruption of mitochondrial fusion or upregulation of mitochondrial fission has been observed in DKD in both podocytes and tubules (178) and are linked to redox imbalance and oxidant stress (179). Targeted deletion of the mitochondrial fission regulating protein Drp-1 in podocytes not only improved DKD but also reduced mitochondrial ROS emission significantly. Targeting these processes can also be therefore beneficial in restoring redox balance besides mitochondrial function while improving DKD outcomes.

## MEASURING REACTIVE SPECIES IN DKD

In addition to a more specific definition of what ROS species is being implicated in DKD, proper detection of reactive species is an important topic. The past decade has seen a rapid improvement in techniques, and the literature offers several state-of-the-art approaches for the detection of redox reactive species. Without going into too much detail, as the topic would deserve a separate review, we would like to provide a snapshot on the applicability in DKD research.

When detecting free radicals, electron spin resonance/EPR is the gold standard approach, combined with spin trapping. The biggest advantage is, as it is spectroscopy, the unambiguous identification of the paramagnetic species, the radical adduct itself instead of fingerprint products or secondary species derived from the free radical reaction. We and others have demonstrated the applicability of the technique in renal cells and kidney tissue, from NO detection to lipid peroxidation (64, 110, 120, 154, 180). The disadvantages are that not everyone has access to EPR, nor it has become widespread in biology research as it requires specific training in biophysics. What are good alternatives and what should be avoided? The literature is extensive on the first generation of various fluorescent dyes (dichlorofluorescein, dihydroethidium, MitoSox, and similar). In principle, these should be used with caution and, in some cases, to be avoided when possible. The main reason is that they either “redox cycle,” meaning they may generate the radical that they are purported to measure (181), or they generate artifacts and are not specific (182, 183). Some, for example, DHE, when combined with HPLC, however, can be appropriate to detect free radicals, because HPLC can distinguish the autoxidation product from the real redox reaction, both of which otherwise may just produce the same red fluorescence (184). The newer, next-generation redox probes now offer much more demonstrated specificity, such as akylboronates for H_2_O_2_ and peroxynitrite detection (185) or, for example, MitoPeDEPP for live cell mitochondrial lipid peroxide detection (186). A further challenge in the field is the in vivo application of such probes as in vivo conditions/factors are very much different from those in vitro. A promising approach may be the use of green fluorescent protein (GFP)-conjugated products to make redox-sensitive probes (such as HyPER or roGFP) (187, 188). Advantages include that calibration with these probes is now possible, the protein is modifiable with sequences to target the probe to various intracellular locations (mitochondria or extracellular matrix), and, last but not least, with cell-specific promoters, transgenic animal models can be generated (117). Finally, with emerging new state-of-the-art mass spectrometry imaging approaches like matrix-assisted laser desorption/ionization imaging or desorption electrospray ionization combined with imaging, it is now possible not only to identify a myriad of oxidized species such as redox phospholipids but also their spatial distribution and location across the kidney (63).

## FUTURE PERSPECTIVES

To summarize, this review aimed to highlight the complex role of redox biology in DKD, in light of the current literature and some of our own views. Previous rigorous and elegant work has laid the foundation of the concepts of redox regulation and imbalance of ROS in DKD. Two novel concepts are emphasized in the present review: reactive (oxidized) phospholipids and their specificity in cell death and cell-cell cross talk in the kidney. Moving toward a more precise and defined understanding of what we mean by “redox signal,” redox regulation, and specifying sources, renal cells, nephron segment areas, and the molecular entity of oxidized species will aid us for more targeted interventions in combatting this complex disease.

## GRANTS

This work was supported by National Institutes of Health Grants DK115749 (to K.S.) and R01DK097253 and E01W81XWH2010836 (to I.S.D.), and by American Heart Association Grant 23PRE1025798 (to U.S.E.).

## DISCLOSURES

I.S.D. has a consultancy agreement with RUMI. None of the other authors has any conflicts of interest, financial or otherwise, to disclose.

## AUTHOR CONTRIBUTIONS

I.S.D. and K.S. prepared figures; I.S.D. and K.S. drafted manuscript; I.S.D., U.S.E., and K.S. edited and revised manuscript; I.S.D., U.S.E., and K.S. approved final version of manuscript.
